# The mechanical responses of advecting cells in confined flow

**DOI:** 10.1063/5.0005154

**Published:** 2020-05-04

**Authors:** S. Connolly, D. Newport, K. McGourty

**Affiliations:** 1 School of Engineering, Bernal Institute, University of Limerick, Limerick V94 T9PX, Ireland; 2 School of Natural Sciences, Bernal Institute, University of Limerick, Limerick V94 T9PX, Ireland; 3 Health Research Institute, University of Limerick, Limerick V94 T9PX, Ireland

## Abstract

Fluid dynamics have long influenced cells in suspension. Red blood cells and white blood cells are advected through biological microchannels in both the cardiovascular and lymphatic systems and, as a result, are subject to a wide variety of complex fluidic forces as they pass through. *In vivo*, microfluidic forces influence different biological processes such as the spreading of infection, cancer metastasis, and cell viability, highlighting the importance of fluid dynamics in the blood and lymphatic vessels. This suggests that *in vitro* devices carrying cell suspensions may influence the viability and functionality of cells. Lab-on-a-chip, flow cytometry, and cell therapies involve cell suspensions flowing through microchannels of approximately 100–800 μm. This review begins by examining the current fundamental theories and techniques behind the fluidic forces and inertial focusing acting on cells in suspension, before exploring studies that have investigated how these fluidic forces affect the reactions of suspended cells. In light of these studies’ findings, both *in vivo* and *in vitro* fluidic cell microenvironments shall also be discussed before concluding with recommendations for the field.

## I. INTRODUCTION

The transport of cells by fluids at the sub-millimeter scale plays a key role in physiology and bioprocessing.

Red blood cells (RBCs), white blood cells (WBCs), and sometimes cancer cells are advected through biological microchannels in both the body’s cardiovascular system (CS) and the lymphatic system (LS) and, as a result, are subject to a wide variety of complex fluidic forces as they pass through. Here, these microfluidic forces influence different biological processes such as the spreading of infection, cancer metastasis,^1^ and cell viability,^2–9^ highlighting the importance of fluid dynamics in the blood and lymphatic vessels.

These forces are not only determined by the channel and fluid properties, but also by the mechanical properties of the cells themselves. The deformability of suspended cells can change due to illness or disease progression,^10–19^ inciting a change in the deformability-induced lift experienced by these cells, which in turn causes a change in the distribution of cells across the channel width^20,21^ which can be exploited in cell separation techniques.^13,22,23^

It is, therefore, likely that the forces experienced by cells flowing in devices may affect both viability and functionality.^24^ Lab-on-a-chip and other microfluidic devices and techniques, such as flow cytometry and cell sorting, involve cell suspensions flowing through microchannels. These techniques are already commonly used in widespread applications ranging from clinical settings to research environments.

At present, the effects that fluid mechanics exert on cells in suspension have largely remained unexamined.^24,25^ Despite the fact that it is desirable for cells to retain their integrity following such diagnostic procedures, it is not considered to be essential. However, the topic is rapidly gathering importance with the recent emergence of cell therapies. Methods such as chimeric antigen receptor (CAR) T-cell therapy or adoptive T-cell transfer therapy, require the removal of cells from the patient, cell processing and reintroduction of the cells to the individual, carried out via microchannels. With the extraction of cells from the bodies of immunologically compromised patients, there exists a critical need to ensure that the viability or functionality of the precious supply of cells has not been compromised.

These findings will be important considerations in the design of such devices for both diagnostic and research purposes, but is of particular concern in cell therapy whereby cells are returned to the body following treatment. Therefore, it is important to understand completely the forces that suspended cells are subject to, both *in vivo* and *in vitro*, as they may affect the viability and functionality of the cells.

This review begins by examining the current fundamental theories behind the fluidic forces and inertial focusing acting on particles, and by extension, cells, in suspension, before exploring studies which have investigated how these fluidic forces affect the reactions of suspended cells. An examination of both the *in vivo* and *in vitro* cell environments shall be discussed in light of these studies’ findings and how the results can be interpreted in such contexts before finally, concluding with recommendations for the field.

## II. THE THEORETICAL MECHANICS OF PARTICLE FLOW

The behavior of fluids and their suspended particles change as the scale of the channel changes from the macroscale to the microscale. Often the behavior of cells, being of somewhat similar size and shape, can be related to suspended particles. Here, the theory behind the behavior of these particles and cells is described as well as the fluids they are suspended in.

### A. Poiseuille flow

The physics of flow in a pipe provide the fundamental theories behind fluid flow in the channels of the body as well as in *in vitro* microfluidic channels. Poiseuille flow, the fully developed, laminar, pressure-driven flow of an incompressible fluid in a circular pipe, can be described using the equation

$$U(r)=2\bar{U}\left(1-{\left(\frac{r}{R}\right)}^{2}\right),$$ (1)

where U(r) is the fluid velocity profile with respect to the channel radial position, r, $\bar{U}$ is the mean velocity, and R is the channel radius, resulting in a maximum fluid velocity at the channel’s center. The shear stress [τ(r)] across the channel width can be defined as

$$\tau (r)=\mu \frac{dU(r)}{dr}=-\frac{4\mu \bar{U}r}{R^{2}},$$ (2)

where τ(r) is the shear stress profile with respect to the channel radial position. This results in the largest τ(r) in the channel at the wall ($\tau_{w}$) and can be expressed as

$$\tau_{w}=\frac{8\bar{U}\mu }{D},$$ (3)

where D is the diameter of the channel. Note that both τ(r) and $\tau_{w}$ are the shear stress values that a particle experiences at a point in the channel due to its radial position, and is not necessarily the shear stress gradient acting on the surface of the particle ($\nabla \tau_{p}$).

The Reynolds number (Re), used to describe the relationship between the inertial and viscous forces in a channel, is defined as

$$Re=\frac{\rho \bar{U}D}{\mu },$$ (4)

where ρ is the density of the fluid. Pipe flows with a Re> 2000 can be described as turbulent flow, while below this figure, the flow is usually laminar. In microchannel flow, due to the small channels and flow rates, the viscous forces dominate and Re is quite low as a result. For this reason, microfluidic flow is usually laminar.^26^

### B. Particle flow

The first studies examining the inertial migration of particles was carried out by Segré and Silberberg in 1962.^27,28^ They found that in a circular pipe, particles formed an annulus at a distance of 0.6 of the channel radius from the channel center. This effect was subsequently named the Segré–Silberberg effect. Interestingly, this effect does not only take place at the macro-level, but also at the micro-level. Subsequent studies have shown that a number of channel properties influence the inertial migration of these particles. The forces that these particles are subject to have also been examined.

#### 1. Channel properties

A number of channel and flow properties influence the inertial migration of particles in a straight, regular channel, including the Reynolds number, particle Reynolds number, the channel focusing lengths, and the channel geometry.

##### 1. Reynolds number

The Reynolds number [as described in Eq. (4)] influences the positions of particles. At low Re, particles focus toward the Segré–Silberberg positions, while at very high Re, they focus at the channel center.^29,30^ Interestingly, despite the strong influence that Re has on the inertial positions of rigid particles, computational studies have shown that Re has little to no impact on the migration of deformable particles, or cells, in circular channels.^31^

##### 2. Particle Reynolds number

The particle Reynolds number ($Re_{p}$) is one of the main descriptors of particle behavior in a fluid flow. $Re_{p}$ in a circular pipe is defined as

$$Re_{p}=Re{\left(\frac{d_{p}}{D}\right)}^{2},$$ (5)

where $d_{p}$ is the diameter of the particle. The $Re_{p}$ can be used to describe the point at which the inertial migration of particles begins to take place. Previous experimental studies have shown that $Re_{p}$ needs to have reached a value of at least 0.05 with a particle to diameter ratio ($\frac{d_{p}}{D}$) of at least 0.07 in order for particle migration to have occurred.^32^

##### 3. Channel length

An appropriate channel length is required in order for particles or cells to reach equilibrium Positions; however, there is no consensus among microfluidic experts on a single equation and several formulas have been proposed in order to calculate this length.^33^ Bhagat *et al.*^32^ proposed that

$$L_{M}=\frac{3\pi \mu }{2\rho \bar{U}}{\left(\frac{d_{p}}{D}\right)}^{3},$$ (6)

where $L_{M}$ is the migration length. This equation was also used by Zhang *et al.*^34^ and Gao.^33^ Di Carlo^26^ used the following expression:

$$L_{M}=\frac{\pi \mu D^{2}}{\rho U_{max}{d_{p}}^{2}C_{L}},$$ (7)

where $U_{max}$ is the maximum velocity of the fluid (normally at the channel center) and $C_{L}\approx$ 0.04 with a channel aspect ratio of 1 ($C_{L}$ varies from approximately 0.02–0.05 as the channel aspect ratio ($\frac{H}{W}$, where H is the channel height and W is the channel width) varies from 2 to 0.5). This expression was favored by Amini *et al.*^35^ and Martel and Toner.^36^

##### 4. Channel geometry

**FIG. 1.**
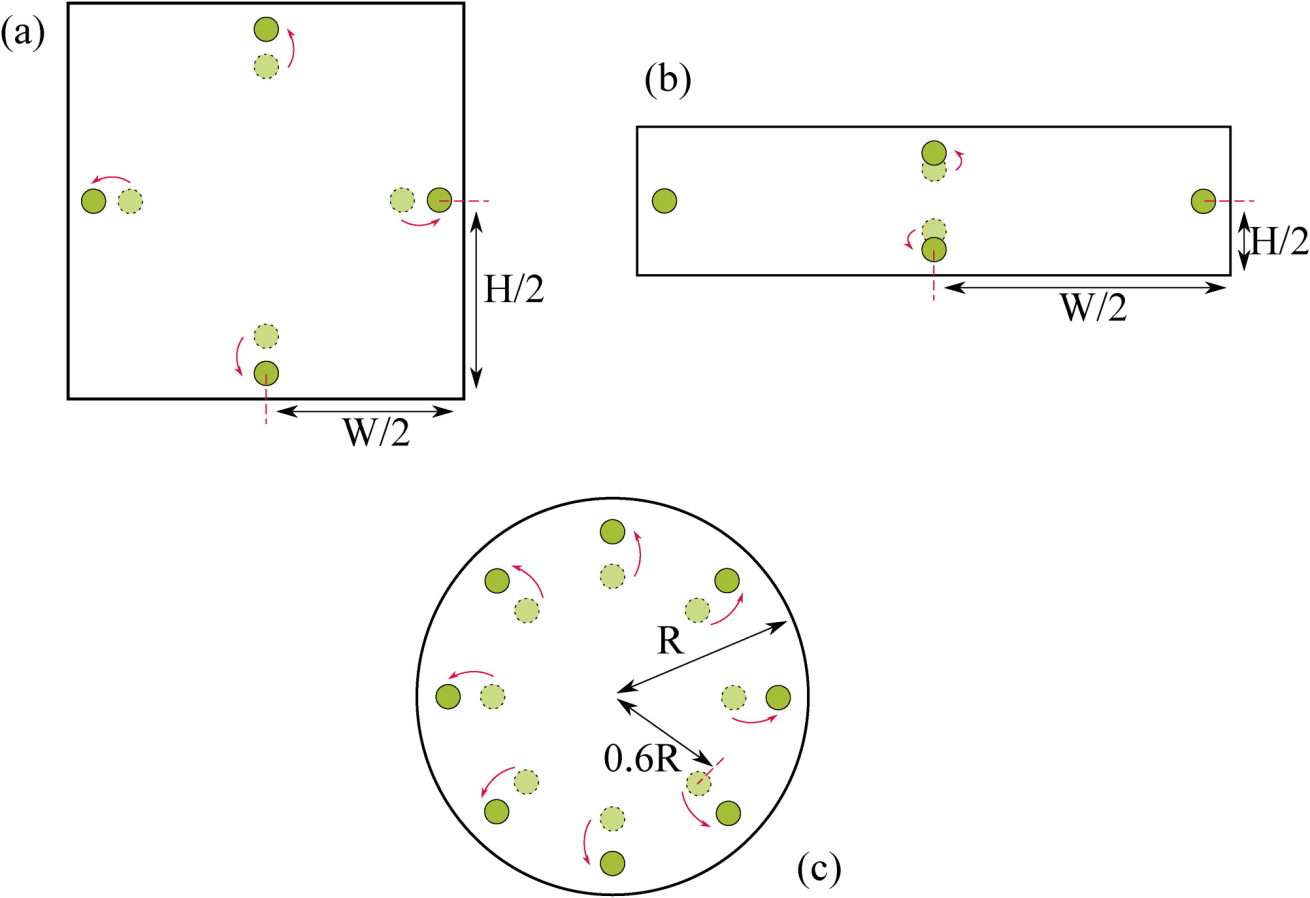
Particle equilibrium positions in different channel geometries in (a) a square channel, (b) a rectangular channel, and (c) a circular channel. As the flow rate increases, particles migrate to positions in the directions of the arrows.

The geometry of the channel significantly affects the equilibrium positions of particles and cells alike. As previously described, in circular channels, particles form an annulus at 0.6 of the channel radius. In square channels, particles equilibrate at the center of the channel walls as seen in Fig. 1. An increase in the flow rate results in particles moving closer to the walls. In rectangular channels, the particles equilibrate at the center of the two larger walls. This can also be seen in Fig. 1. In this instance, as the flow rate is increased, particles at the longer edge move toward the walls, similar to square channels, while particles also start to form equilibrium positions at center of the shorter two walls.^26,32–43^ As this study focuses primarily on circular microchannels, the behavior of particles in channels of different geometries is not explored in great detail here. Further reading on the topic is available at the above references.

#### 2. Particle forces

**FIG. 2.**
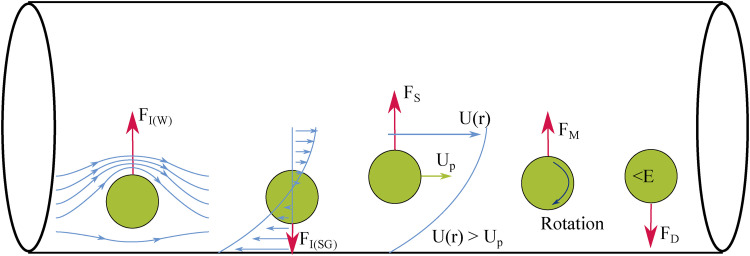
Illustration of the lift forces acting on a particle in microchannel flow.

Both particles and cells flowing in a microchannel have normal and shear stresses acting on their surfaces. These can be divided into lift forces (due to normal stresses) and drag forces (due to shear stresses). Lift forces include the wall-induced lift, shear-gradient-induced lift, Saffman (or slip-shear-induced) lift, Magnus (or rotation-induced) lift, and deformability-induced lift, while drag forces include viscous drag. The lift forces are illustrated in Fig. 2.

##### 1. Inertial lift force

The main force acting on a particle is the inertial lift force ($F_{I}$), which also plays a significant part in cellular focusing. This is a balance between the wall-induced lift ($F_{I(W)}$), and the shear-gradient-induced lift [$F_{I(SG)}$], as shown in Fig. 2. When a particle flows close to the channel wall, fluid streamlines around the particle are directed to its opposite side. This creates a pressure difference between the two sides of the particle, causing it to be directed toward the channel center by $F_{I(W)}$. This can be expressed as $F_{I(W)}\propto \frac{\rho {\left(U_{max}\right)}^{2}{d_{p}}^{6}}{D^{4}}$. Conversely, $F_{I(SG)}$ opposes $F_{I(W)}$. As previously discussed, in Poiseuille flow, the velocity profile is parabolic. Due to this, the fluid velocity relative to the particle velocity will be larger on the wall side of the particle than on the side closer to the channel center. This generates $F_{I(SG)}\propto \frac{\rho {\left(U_{\max}\right)}^{2}{d_{p}}^{3}}{D}$, which directs the particle toward the channel wall.^26,32–36,38–49^ $F_{I}$ was derived using the matched asymptotic expansion method by Asmolov^46^ to be

$$F_{I}=\frac{\;f_{L}\rho {\left(U_{max}\right)}^{2}{d_{p}}^{4}}{D^{2}},$$ (8)

where $f_{L}$ is the lift coefficient. This was completed by matching the inner and outer flow region pressures and velocities. This equation holds true as long as the particle size is much smaller than the size of the channel ($\frac{d_{p}}{D}\ll 1$). As $F_{I}\propto {d_{p}}^{4}$, larger cells have much larger $F_{I}$ acting on them, which could have potential implications for cell viability.

##### 2. Saffman lift force

As a particle flows in a microchannel, drag forces from the wall act on it, causing it to travel at a lower velocity than the fluid it is suspended in. In this case, a force due to the slip-shear, the Saffman lift force ($F_{S}$), causes the particle to migrate toward the side of the maximum relative velocity. This is illustrated in Fig. 2. In other words, if a particle is lagging the flow, $F_{S}$ will direct the particle toward the channel center; however, a particle leading the flow will be directed toward the wall. It is worth remembering that in general, $F_{S}$ in a channel will be an order of magnitude smaller than $F_{I}$ and so can be considered negligible.^33–36,42,48,50–52^ $F_{S}$ was also derived using the matched asymptotic expansion method by Saffman,^50^

$$F_{S}=KV\frac{{d_{p}}^{2}}{4}{\left(\gamma \nu^{-1}\right)}^{\frac{1}{2}},$$ (9)

where K is a constant (approximately 81.2), V is the relative velocity between the fluid and the particle, γ is the velocity gradient, and ν is the kinematic viscosity. This was completed by matching the inner and outer velocity expansions. In the context of microchannels, cells tend to lag the flow;^20^ therefore, though the effect from $F_{S}$ will be small, it will direct cells toward the channel center.

##### 3. Magnus lift force

A particle in microchannel flow will also experience a degree of rotation. Due to the difference in the fluid velocity on either side of the particle, it will rotate in the flow as seen in Fig. 2. Rotation in the counterclockwise direction, as indicated in the schematic, results in a pressure difference on either side of the particle with the lower pressure on the side of the larger fluid velocity (in this case it is the upper side). This causes a force due to the rotation, the Magnus force ($F_{M}$), to act in the direction of lower pressure. In the case of microchannel flow, this is generally toward the channel center. $F_{M}$ is also very small (usually an order of magnitude less than $F_{S}$) in comparison to other lift forces, and so can be deemed negligible; however, its effects increase significantly the closer the particle is to the wall. $F_{M}$ also scales with the cube of $d_{p}$.^33,34,41,42,51–54^ $F_{M}$ was derived using the matched asymptotic expansion method by Rubinow and Keller,^53^

$$F_{M}=\frac{1}{8}\pi {d_{p}}^{3}\rho (\vec{V}\times \vec{\Omega }),$$ (10)

where $\vec{V}$ is the relative velocity vector between the particle and the fluid and $\vec{\Omega }$ is the angular velocity vector of the particle. This was carried out by matching the Stokes and Oseen expansions.

##### 4. Deformability lift force

Deformability-induced lift ($F_{D}$) affects non-rigid particles such as cells or vesicles. It has been argued that $F_{D}$ is due to the shape change that deformable particles in a flow experience due to fluidic forces;^55^ however, it can also be attributed to the surface tension gradients at the interface between the fluid and particle surface.^56^ For this reason, the Weber number [ratio of inertial stress to surface tension, Eq. (11)] and the capillary number [ratio of viscous stress to surface tension, Eq. (12)], which both describe the surface tension gradient, along with the viscosity ratio (between the particle and fluid viscosities, Eq. (13)), which describes the particle shape, are important. As these numbers can be used to characterize cell or droplet deformation, which increases with $F_{D}$, they can be utilized to estimate its scale.^23^ The Weber number (We) can be defined as

$$We=\frac{\rho {\bar{U}}^{2}d_{p}}{\sigma },$$ (11)

where σ is the surface tension. The capillary number (Ca) can be defined as

$$Ca=\frac{\mu \bar{U}d_{p}}{\sigma D}.\;$$ (12)

Finally, the viscosity ratio ($\lambda_{p}$) can be defined as

$$\lambda_{p}=\frac{\mu_{p}}{\mu },$$ (13)

where $\mu_{p}$ is the viscosity of the particle’s internal fluid. Under Poiseuille flow, it has been found that a more deformable particle (or one with a low elastic modulus (E)) migrates toward the channel center, while stiffer, more rigid particles remain closer to the channel walls.^23,34,35,43,55–58^ This can be seen in Fig. 2. The deformability-induced lift was derived by Chan and Leal^57^ to be

$$F_{D}=Ca\mu \bar{U}d_{\;p}{\left(\frac{d_{\;p}}{D}\right)}^{2}\left(\frac{r}{D}\right)f(\lambda_{\;p}),$$ (14)

where r is the radial position of the particle in the channel, and $f(\lambda_{p})$ is defined as

$$f(\lambda_{p})=\frac{128\pi }{{\left(\lambda_{p}+1\right)}^{3}}\left[\frac{11\lambda_{p}+10}{140}(3{\lambda_{p}}^{2}-\lambda_{p}+8)+\frac{3(19\lambda_{p}+16)}{14(3\lambda_{p}+2)}(2{\lambda_{p}}^{2}-\lambda_{p}-1)\right],$$ (15)

given that the particle is not too close to the channel walls ($R-r>d_{p}$) and that $1<\lambda_{p}<10$.^35,57^ Based on their experiments, Stan *et al.*^58^ then derived an equation for the empirical inertial lift force ($F_{I(D)}$) on deformable particles

$$F_{I(D)}=C_{L}\mu \bar{U}d_{p}{\left(\frac{d_{p}}{D}\right)}^{3}\left(\frac{r}{D}\right),$$ (16)

where $C_{L}$ is the lift coefficient, which needs to be experimentally determined for a given combination of continuous and dispersed phases.^34,35,58^ The deformability of the particle also heavily influences its migration speed, with more deformable particles reaching equilibrium positions quicker.^31^

In some instances, when the deformability-induced lift is larger than those generated by cell or droplet deformation, the excess lift may be due to Marangoni-like effects.^58^ The redistribution of surfactants on the surface of the cell induced by thermal gradients or uneven shear rates can cause a flow at the interface between the cell and suspending liquid in the direction of higher surface tension. As the Marangoni effects affect the distribution of σ on the cell surface rather than the absolute value of σ, it does not impact on Ca. However, like Ca, their magnitude depends on the presence and amount of surfactant.^58^

###### 1. Cell deformability

Different cell types within the body vary widely in their mechanical properties, including in their deformability. This is also true for cells, which are ordinarily suspended in confined flow, primarily RBCs, WBCs, and circulating tumor cells (CTCs). RBCs have a bi-concave disk shape and are very small in comparison to other circulating cells, typically sized approximately 5–9 μm,^13,22,59–69^ and make up approximately 41% of the total blood volume. They are highly deformable in order to squeeze through the narrowest capillaries and previous studies that have examined the deformability of RBCs are outlined in Table I.

**TABLE I.** Deformability studies of red blood cells.

RBC stiffness	Donor	Measurement technique	Study
Approximately 650 *μ*Pa	Human	Micropippette aspiration	10
320 ± 50 *μ*Pa	Human	Laminar flow system	11

The size and shape of WBCs differ between types; however, the majority are in the region of 8–20 μm in diameter.^64,66,70,71^ WBCs make up less than 4% of the total blood volume and are also present in lymph, as opposed to RBCs, which are only found in blood. In general, WBCs have been found to be less deformable than RBCs; however, the ability of WBCs to deform has been found to be connected to stronger adhesions with vessel endothelial cells.^72^ Previous studies investigating these deformabilities are outlined in Table II.

**TABLE II.** Deformability studies of white blood cells.

WBC stiffness	Donor	Measurement technique	Study
2–3 kPa	Human	Atomic force microscopy	73
85 ± 5 Pa	Human	Parallel plates	74
1.24 ± 0.09 kPa	Human	Atomic force microscopy	75
11.2 ± 5.9 kPa	Mouse	Atomic force microscopy	76

CTCs primarily invade through the circulatory systems (blood or lymph), and it is believed that in the case of solid cancers, up to 80% of tumor dissemination takes place through the lymphatics while only 20% occurs through the vascular system.^77,78^ CTCs tend to be larger than normal cells, measuring approximately 15–25 μm,^4,7,20,63,79–84^ and this characteristic can be exploited in some cell separation techniques (see Sec. V B). Circulating cells are very rare in the blood of cancer patients, occurring at a rate of 1–100 CTCs per $1\times 10^{9}$ blood cells,^8,20,63,80,85,86^ while their numbers in lymph have not yet been investigated.^20^ The stiffness of CTCs vary depending on the source tissue and the metastatic capabilities of the cells themselves. Previous studies examining the deformabilities of two different breast cancer cell lines (MCF-7 and MDA-MB-231 cell lines) are outlined in Table III.

**TABLE III.** Deformability studies of breast cancer cells.

MCF-7 cell stiffness	MDA-MB-231 cell stiffness	Measurement technique	Study
…	182 ± 34.74 Pa	Constricted microchannel	17
3–4.5 kPa	4–6 kPa	Atomic force microscopy	18
36 ± 8 Pa	18 ± 10 Pa	Optical tweezers	19
87.3 ± 47.8 kPa	55.6 ± 20.1 kPa	Atomic force microscopy	16
30.2 ± 15.0 Pa	12.6 ± 6.1 Pa	Optical tweezers	16
285.1 ± 127 kPa	277.3 ± 63.1 kPa	Atomic force microscopy	87
275.2 ± 157.4 kPa	257.5 ± 98.4 kPa	MEMS resonant sensor	87
1.04 ± 0.27 kPa	…	Atomic force microscopy	88
800 ± 20 Pa	500 ± 25 Pa	Atomic force microscopy	89
…	0.40 ± 0.22 kPa	Atomic force microscopy	90
300–450 Pa	…	Atomic force microscopy	91

As can be seen here, the discrepancies between different studies can be quite varied. Different measurement techniques, as well as different cell media and substrate, can influence the mechanical properties of the cell.^19,90^ It has also been found that a lower E, or softer cell, means the cancer cell no longer firmly fits in its position in the matrix while it is surrounded by stiffer endothelial cells, allowing the cell to detach and migrate more easily.^89^ Despite these discrepancies, however, all studies agree with each other; the more metastatic a cell is, the lower its E and the more compliable it is.^84,92–96^

##### 5. Viscous drag force

When a particle flows through a fluid or a fluid flows past a particle, shear stresses are introduced, resulting in viscous drag forces ($F_{V}$). This can be expressed as

$$F_{V}=\frac{\pi {d_{p}}^{2}f_{drag}}{4},$$ (17)

where $f_{drag}$ is the drag coefficient.^34,42^ The drag coefficient has different definitions depending on the $Re_{p}$. For $10^{-4}<Re_{p}<0.2$,

$$f_{drag}=\frac{12\mu V}{d_{p}}.$$ (18)

This results in a $F_{V}$ of

$$F_{V}=3\pi \mu d_{p}V$$ (19)

or Stokes drag.^34^ The Dean drag force is imposed on a particle due to Dean flow, also known as secondary flow, acting perpendicular to the direction of the main flow. The Stokes drag force scales linearly with particle size and secondary flow velocity and inversely with the channel’s radius of curvature. Therefore, in straight channels, the Dean drag force can be considered to be negligible.^26,33,35,36,41–43,47^ For $0.2<Re_{p}<500\text{–}1000$,

$$f_{drag}=\frac{12\mu V}{d_{p}}(1+0.15{Re_{p}}^{0.687}),$$ (20)

resulting in a $F_{V}$ of

$$F_{V}=3\pi \mu d_{p}V(1+0.15{Re_{p}}^{0.687})$$ (21)

(Refs. 34 and 97). Finally, for $500\text{–}1000<Re_{p}<2\times 10^{5}$,

$$f_{drag}=0.22\rho V^{2}$$ (22)

resulting in a $F_{V}$ of

$$F_{V}=0.055\pi {d_{p}}^{2}\rho V^{2}$$ (23)

(Ref. 34). In this way, the $F_{V}$ is influenced both in the flow direction by the mainstream flow and in the lateral direction by the secondary flow.^34^

All of the particle forces are summarized in Table IV

**TABLE IV.** Summary of lift and drag forces acting on a particle in microchannel flow.

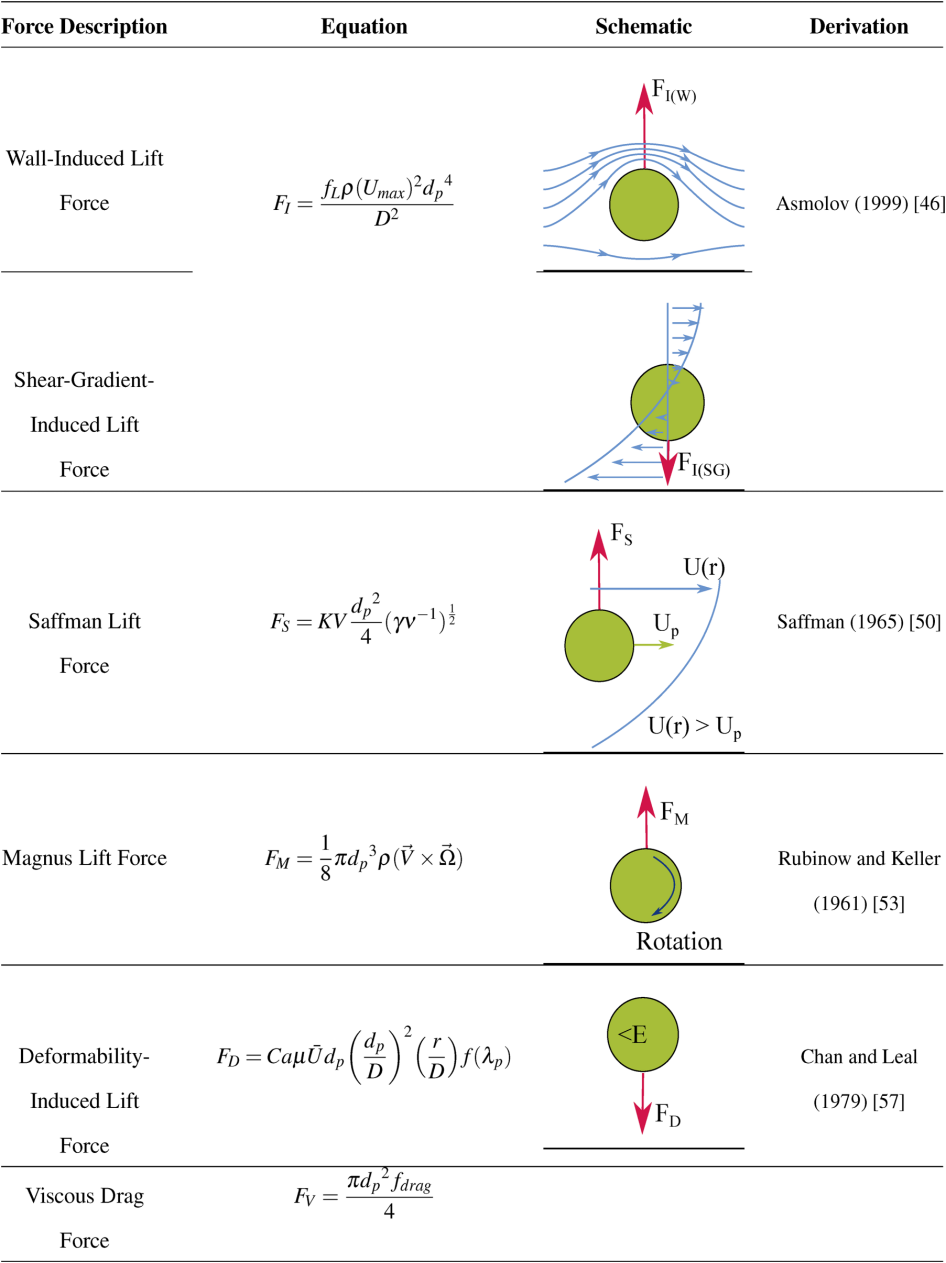

## III. THE IMPACT OF THE MICROFLUIDIC ENVIRONMENT ON SUSPENDED CELLS

As shown in Sec. II B, the fluid mechanics in the microchannel environment have a significant impact on the forces that solid suspended particles are subject to, and consequently, their inertial positions. Therefore, it is not unreasonable to assume that these same forces will impact suspended cells flowing in a microchannel, be it *in vivo* or *in vitro*. As cells are living organisms, the microfluidics in the channel can affect more than just their trajectories within the channel. Fluidic forces have been shown to influence the viability or functionality of living cells,^6^ while they may also influence cell behaviors such as cell adhesion to the vessel wall.^86^

### A. Cell viability

Due to the extremely low efficiency of the process of metastasis (less than 0.01% of CTCs form a tumor at a secondary site^7–9,85,98–101^), many researchers hypothesize that high τ(r) in the vasculature can cause cell death.^99,102–107^ Contrastingly, others argue against this conjecture that CTCs are not mechanically fragile and have developed certain mechanisms to prevent damage from τ(r) in the circulatory environment.^8,86,108,109^ Furthermore, they argue that certain levels of τ(r) in fact promotes CTC invasion^110–113^ and can simulate the growth of cancer cell clusters.^114^ A number of studies investigating the proposition have been conducted through different methods and are outlined below.

#### 1. Cone and plate experiments

**FIG. 3.**
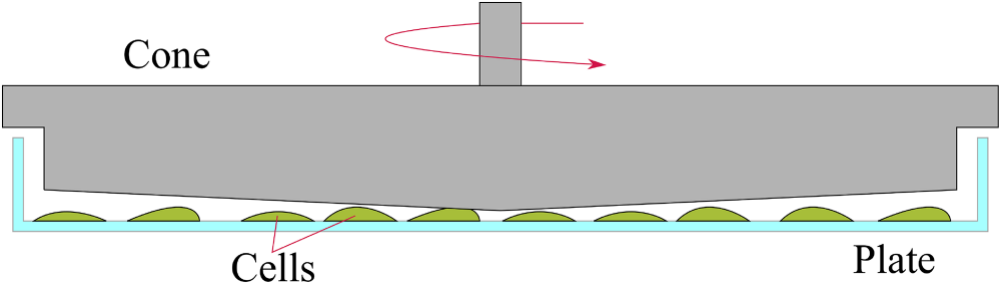
A cone and plate setup applies a constant shear stress profile to adherent cells.

A cone and plate viscometer can be used to replicate Couette flow by applying a uniform, consistent τ(h) to cells which are adherent to a plate^115^ (see Fig. 3). This method has been used by different studies to examine the viability of cancer cells under constant flow conditions, finding that the viability of B16 melanoma cells is reduced to 0 after exposure to τ(h) levels of 2.9 Pa for 5.5 h, while ovarian cancer cells exposed to τ(h) levels of 1.2 Pa had reduced viability by 30% after 10 min.^8^ Further studies have found that exposure of breast cancer cells to 6 Pa for a 24 h period results in a cell viability of close to 0.^21^ These studies all conclude that cells exposed to a threshold τ(h) level over a considerable time frame will result in cell death. However, while useful for applying a constant, known τ(h) value, the cone and plate setup does not mimic the pipe-imposed shear that suspended cells would ordinarily be exposed to *in vivo* or *in vitro*.

#### 2. Syringe and needle experiments

The syringe and needle method, consisting simply of a suspended cell solution, injected via a syringe through a needle of known diameter, is one of the simplest methods for replicating Poiseuille flow (see Sec. II B). In the implementation of this method, experiments typically last seconds, rather than hours. For this reason, very high $\tau_{w}$ values were examined. It was found in several studies that $\tau_{w}$ values of approximately 600–640 Pa resulted in cancer cell viability of 50%–80% following 10 min of exposure.^2,3,109^ Others have found that under lower $\tau_{w}$ values (2–6 Pa), cancer cell viability was unaffected.^116^ Additionally, it has been found that 450–560 Pa applied to RBCs is enough to rupture their membranes.^8^ The studies indicated that non-transformed cells were not able to withstand such high $\tau_{w}$ values that cancer cells were, indicating that CTCs possess a property of cellular transformation that provides them with a certain resistance to τ(r), protecting them *in vivo*.

#### 3. Continuous flow circuits

Continuous flow circuits, comprising a peristaltic pump, circulating suspended cells around a flow circuit, have also been used. While this model more closely represents the pulsatile flow and resulting $\tau_{w}$ that cells are exposed to in the CS, it fails to entirely capture the fluid dynamics of the CS.^8^ Additionally, peristaltic pumps may also impose additional forces on cells, causing cell death to be incorrectly attributed to the wall shear stresses in the tubing.^21^ $\tau_{w}$ values are orders of magnitude lower than those experienced in the syringe and needle experiments, while viability rates in these systems are much lower than those observed in the cone and plate experiments, with periodic, pulsatile exposure to $\tau_{w}$ of approximately 6 Pa reducing the viability of suspended cells down to only 20%, or even less in some cases, over 18–24 h.^5–7^ In other cases, $\tau_{w}$ of 3 Pa over 24 hrs had a similar effect,^4^ while a $\tau_{w}$ of 2 Pa over 12 h reduced cell viability to below 40%.^9^ Further studies have found that $\tau_{w}$ of 1.6 Pa over 12 h resulted in a viability level of approximately 40%; however, cells in suspension, but not circulation, over the same period of time resulted in similar viability levels, suggesting that cell death may be attributed to anoikis rather than being solely due to the fluidic conditions in the channel.^25^ Interestingly, the same experimental setup has been used to demonstrate that certain levels of shear stress (approximately 3 Pa), below that which will cause a decrease in cell viability, acts as a stimulant for cancer cell migration,^117^ while shear stress on circulating cells can enhance certain anti-cancer drugs.^118^

In both the syringe and needle methods and continuous flow circuits, due to the fact that the cells are in suspension, it is difficult to determine the actual shear that the cells were subjected to. For this reason, while utilizing both of these experimental setups, $\tau_{w}$ was calculated using Eq. (3) and this was assumed to be the shear stress that the cells were exposed to in the channel. However, the cells would only experience these levels of stress if they were traveling at the wall and do not perturb the flow. Therefore, from a viability perspective, it is necessary to know where the cells are located if the local fluid Poiseuille shear stress is to be estimated.

### B. Cell inertial positions

A large number of studies have been carried out on the inertial positions that cells occupy in microchannel flow, replicating conditions that cells may be exposed to in *in vitro* microenvironments or lymphatic conditions. Particle focusing does not occur in turbulent, pulsatile flow, and so these regimes remain unstudied in this context. Cell behavior differs from particles due to their large size distribution^119^ and deformability. The locations of the cells within the channels are the key to understanding the forces that the cells are subjected to, particularly τ(p), if Poiseuille flow is to be assumed. Numerous techniques have been employed to investigate these inertial effects, including particle streak imaging, particle density measurements and particle tracking. These are explored in further detail below. For all of the following techniques in microfluidics, fluorescent particles or cells flowing in a microchannel are illuminated using a laser or an LED, and visualized using an inverted microscope and attached camera.

#### 1. Particle streak imaging

**FIG. 4.**
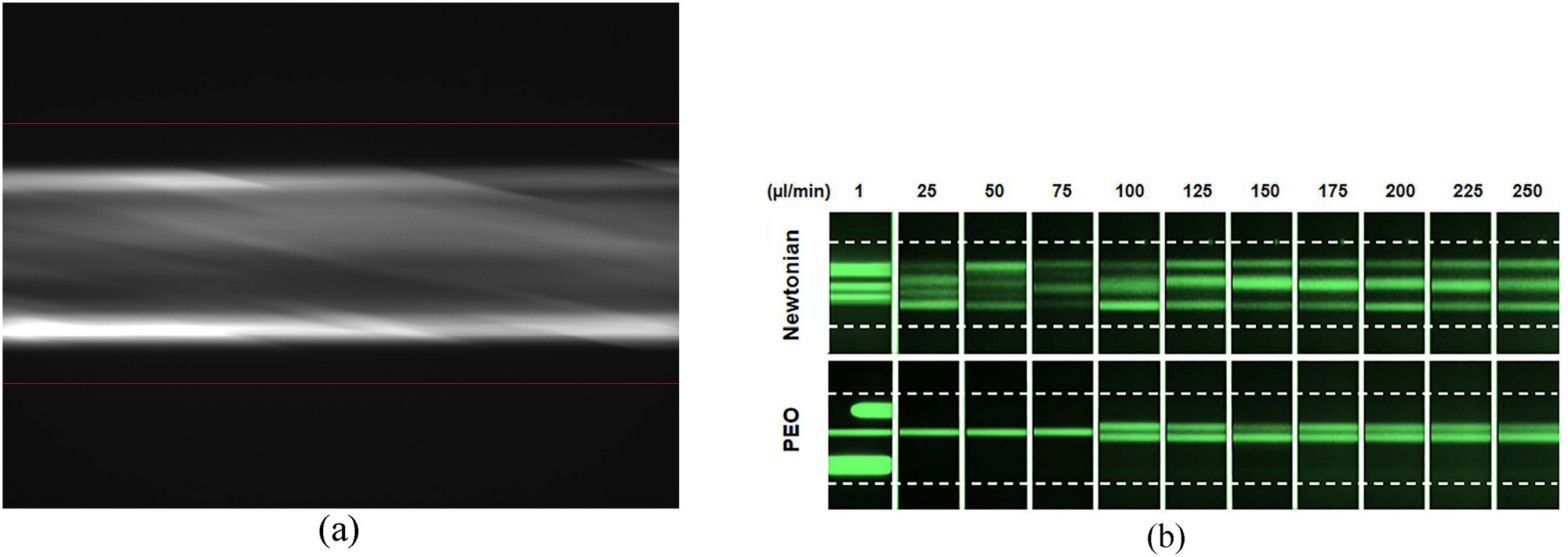
Examples of particle streak images: (a) 30 μm fluorescent particles flowing in a 300 μm inner diameter (ID) circular microchannel at $9.72\times 10^{2}$ μl/min, red lines represent the channel walls, and the exposure time was set to 10 ms. (b) 10 μm fluorescent particles flowing in a 75 μm square microchannel, white dotted lines represent the channel walls, and the exposure time was set to 800 ms. Reproduced with permission from Raoufi *et al.,* Biomicrofluidics **13**, 13 (2019). Copyright 2019 AIP Publishing LLC.

One of the most common, fundamental techniques for assessing particle migration is particle streak imaging. This can be carried out simply by focusing a microscope at the channel center and overexposing the shutter of the attached camera, creating a streak image. Brighter areas imply greater particle densities. Examples of streak images can be seen in Fig. 4. These can be used as standalone images or the image intensities can be averaged over a number of similarly obtained graphics. They are one of the most straightforward methods of assessing the inertial positions of particles.

Particle streak imaging has previously been used in a number of different studies to assess particle positions in a microchannel. Di Carlo *et al.*^38^ used it in order visualize particle focusing in curved microchannels, while Bhagat *et al.*^32^ and Liu *et al.*^120^ also used streak imaging to determine the different inertial positions of particles under different $Re_{p}$ in a square and rectangular microchannel respectively. Additionally, Bhagat *et al.*^32^ visualized particle separation (which will be explored in further detail in Sec. V B). Zhou and Papautsky^41^ were able to use it to calculate the migration distances and focusing lengths of particles and, therefore, the lift coefficient on the particles. The effects of viscosity, cross-sectional channel shape and flow rate were demonstrated by Raoufi *et al.*^121^ using streak imaging [see Fig. 4(b)]. Finally, streak imaging has also been used in biological applications in order to observe DNA focusing and particle focusing in blood in microchannels.^71,122^ Particle streak imaging is useful for small particle sizes in relation to the channel width and is advantageous in that minimal image post-processing steps are required. Experimental time frames are also generally very short (in the order of seconds); however, this could also be construed as a disadvantage.

#### 2. Particle density measurements

**FIG. 5.**
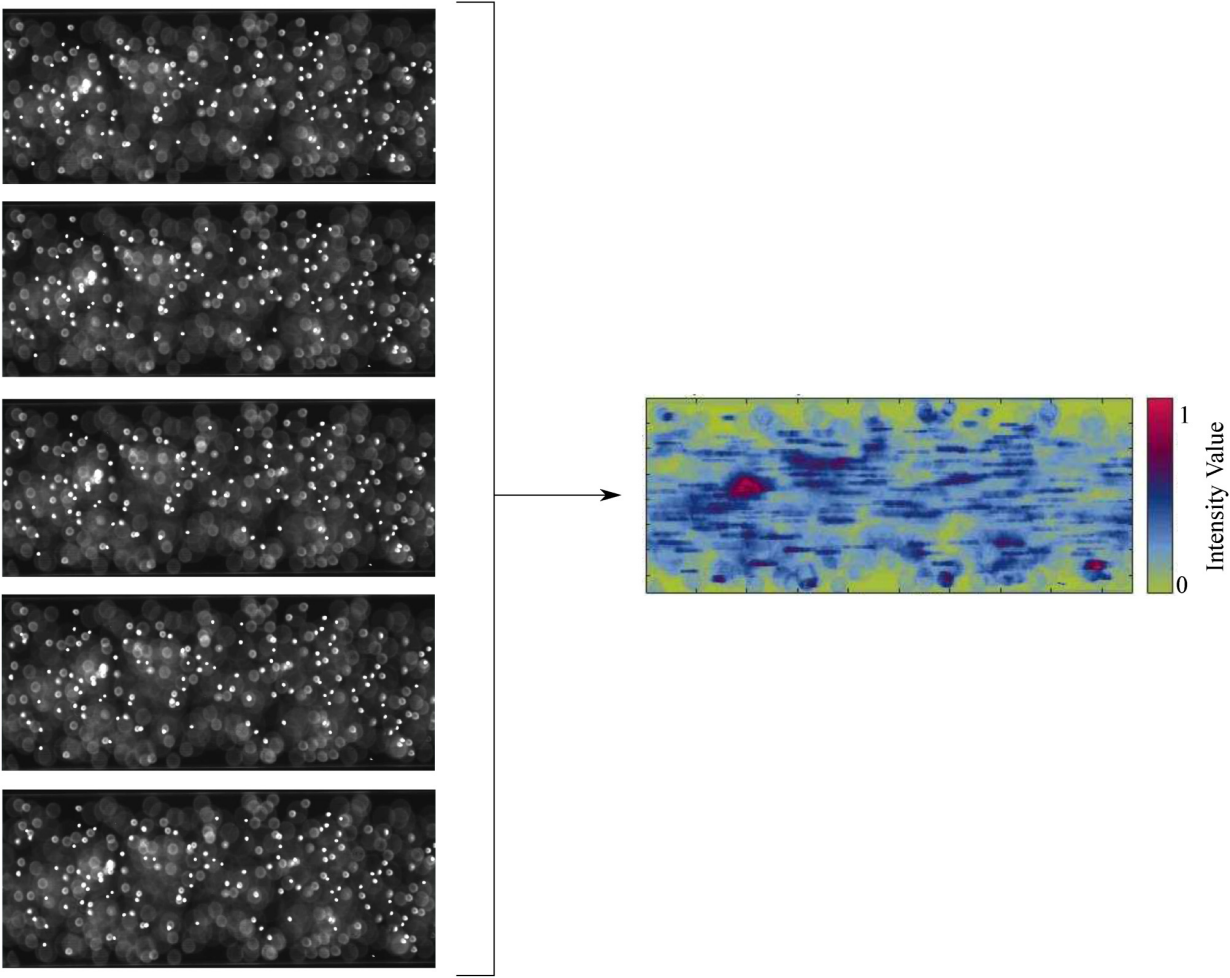
An example of particle density images: a number of images are stacked producing an intensity image of the particle distribution of 10 μm particles within 800 μm ID circular channels.

Like streak imaging, particle density measurements are an Eulerian method of evaluating the inertial positions of particles. This involves taking short exposure images at large intervals. The particle centers are identified and stacked on one image. An example of this can be seen in Fig. 5. Following this, the probability density function (PDF) can be used to evaluate the spatial distribution of the particles in the radial direction. The PDF can be defined as

$$\mathrm{PDF}=\frac{\sum_{i=1}^{I}N_{i}(r,r+dr)}{\sum_{r=0}^{R}\sum_{i=1}^{I}N_{i}(r,r+dr)},$$ (24)

where I is the number of images in the sequence, $N_{i}(r,r+dr)$ is the number of particles between the radial positions r and dr, and R is the radius of the channel.^29,82^

Particle density measurements have previously been used in a number of different studies to assess particle locations. This technique has primarily been used to investigate particle migration under different Re,^29,33,123–131^ and different particle shape.^132^ Park *et al.*^133^ used particle density measurements in order to observe particle distribution in a multi-orifice microfluidic channel, while, again, like streak imaging, particle density measurements have been used in biological applications with Tanaka *et al.*^82^ investigating the inertial migration of cancer cells in blood, and Kulasinghe *et al.*^134^ investigating the migration of cancer cells and cell clusters in square microchannels. Particle density measurements are useful for high density flows, containing small particles in relation to the channel width. Though not as simplistic as streak imaging, post-processing is still less computationally complex than particle tracking. Sampling windows are usually larger (in the order of minutes), making them more accurate than the streak imaging process.

#### 3. Particle tracking

**FIG. 6.**
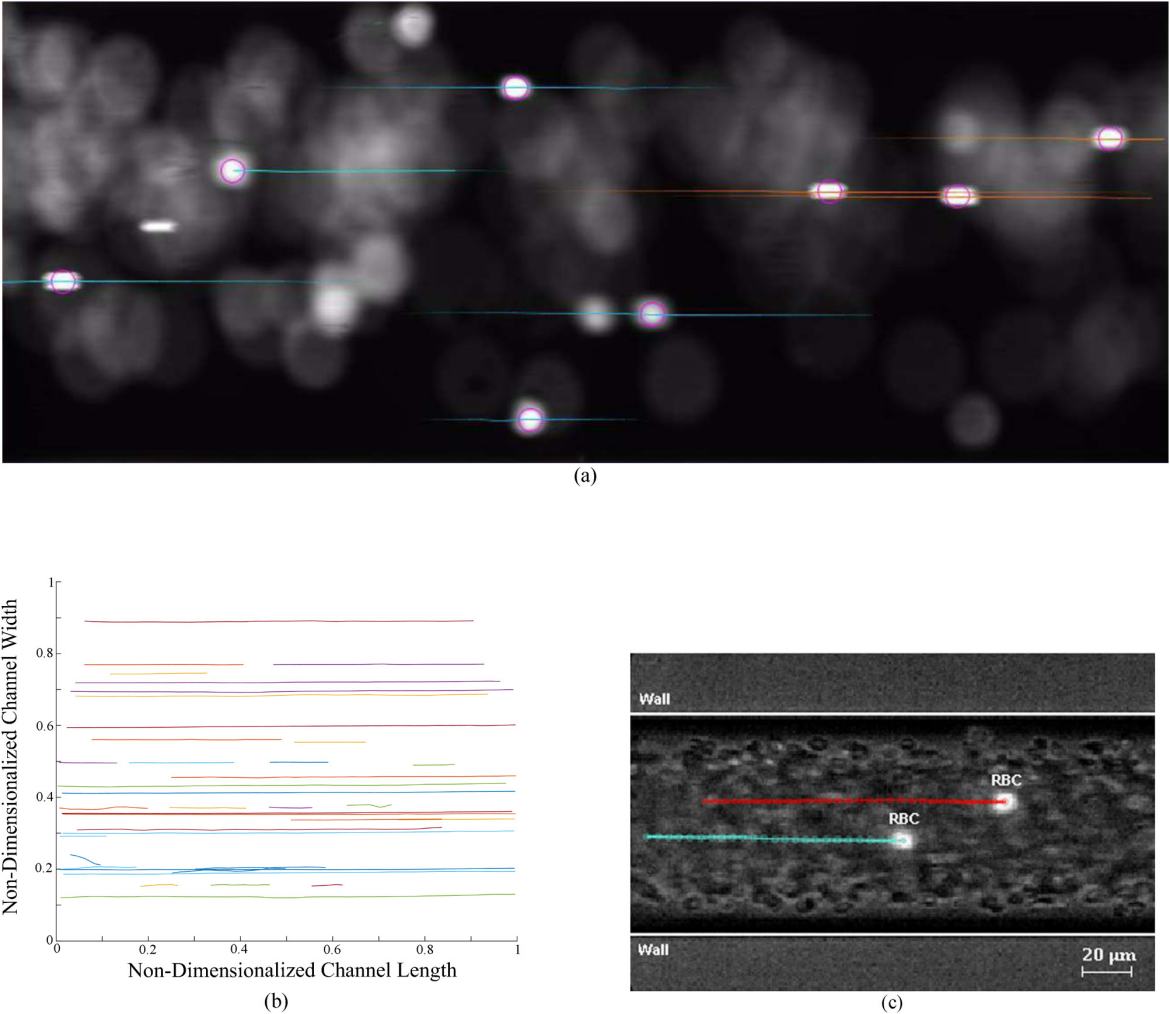
Examples of particle tracking: (a) particle tracking image still of 30 μm fluorescent particles flowing in an 800 μm ID circular channel at 45.3 μl/min, analyzed using the *ImageJ* plugin, *TrackMate*.^137^ (b) All the particle tracks in the same image sequence as (a) collated over 500 images and analyzed using a MATLAB^®^ script. (c) Particle tracking image still of RBCs flowing in a 100 μm ID circular channel, analyzed using *ImageJ* plugin, *MTrackJ*. Reproduced with permission from Pinho *et al.*, J. Biomech. **49**, 2293 (2016). Copyright 2016 Elsevier.

Particle tracking can be used in conjunction with particle tracking velocimetry (PTV). This Lagrangian method involves tracking a particle over the length of the channel. The particle’s average lateral position, with respect to the channel center, over the observation window is measured which is then repeated for many particles, using the equation

$$D_{yy}(t)=\frac{1}{N_{i}}\sum_{n=1}^{N_{i}}\frac{\left[{\left(R_{n,y}(t)-R_{n,y}(0)\right)}^{2}\right]}{2t},$$ (25)

where $D_{yy}$ is the dispersion coefficient, t is the time duration, $N_{i}$ is the number of tracked particles, and $R_{n,y}$ is the radial displacement of the particles.^135,136^ Examples of particle tracking are shown in Fig. 6.

Particle tracking has previously been utilized in order to examine the effects of bidisperse solutions on particle focusing,^138^ as well as to determine the lateral migration of RBCs in 50, 75 and 100 μm ID circular microchannels^135,136,139^ [see Fig. 6(c)] and cancer cells in square microchannels.^20^ Particle tracking is more useful in low density flows, with a large particle to channel size ratio. Image post-processing steps are slightly more complex than those of particle density measurements; However, there is a range of freely available software for such purposes. Unlike streak imaging and particle density measurements, sampling frames are dependent on the flow rate in the channel.

Experimental studies, which have investigated deformable particles flowing in microchannels, are outlined in Table V and are primarily carried out in square or rectangular cross sections, while some have also been completed in circular cross sections. Fewer studies have examined the migration of deformable particles in pipe flow, as opposed to rigid particles, and so, data are only available over a limited range. Furthermore, those experiments conducted at lower Re mainly focus on RBCs, causing non-Newtonian pipe flow. While a large range of studies have investigated the inertial effects of rigid particles under different viscoelastic conditions,^128,140–144^ fewer have examined the same reactions of cells. However, it has been shown that cell suspensions in viscous fluids focus toward the channel center due to elastic forces at the wall.^68^

**TABLE V.** Summary of the studies on inertial migration and particle focusing on deformable particles in microchannels. *Q* is the fluid flow rate and *D*_*h*_ is the hydraulic diameter of the channel.

Particle type	*d*_*p*_ (*μ*m)	Channel description	*Re*/*Q*	Study
Cells—head and neck cancer cells	15	50 × 150 *μ*m^2^ rectangular cross section—20 mm long	*Q* = 100–200 *μ*l/min	134
Cells—yeast cells (*Saccharomyces cerevisiae*)	3–5	75 × 75 *μ*m^2^ square cross section—5 cm long, 60 × 100 *μ*m^2^ rectangular cross section—5 cm long, 75 *μ*m *D*_*h*_ trapezoidal cross section—5 cm long, 75 *μ*m complex cross section—5 cm long	*Q* = 1–250 *μ*l/min	121
Cells—breast cancer cells (MCF-7, MDA-MB-231)	14, 18	100 × 100 *μ*m^2^ square cross section—58.5 mm long	*Re* = 0.02–25	20
Cells—red blood cells	N/A	100 *μ*m ID circular cross section	*Re* = 0.007	136
Macromolecules—DNA	0.5–1	5 × 5 *μ*m^2^ square cross section—4 cm long broadening at 45^°^ to 5 × 50 *μ*m^2^ rectangular cross section	*Re* = 0.11–0.33	122
Cells—white blood cells, prostate cancer cells (PC-3)	9, 17.8	93 × 45 *μ*m^2^ rectangular cross section—3.5 cm long	*Re* ≤ 158	71
Cells—breast cancer cells (MDA-MB-231)	15	220 × 80 *μ*m^2^ rectangular cross section—0.5–2 cm long broadening to 220 × 450 *μ*m^2^ rectangular cross section, 270 × 70 *μ*m^2^ rectangular cross section—1.5 cm long broadening to 270 × 400 *μ*m^2^ rectangular cross section	*Q* = 64–256 *μ*l/min	82
Cells—red blood cells	7	350 *μ*m ID circular cross section	*Q* = 17.3 *μ*l/min	60
Cells—white blood cells	8–15	10 × 25 *μ*m^2^ rectangular cross section—5 mm long, 10 × 50 *μ*m^2^ rectangular cross section—5 mm long, 10 × 75 *μ*m^2^ rectangular cross section—5 mm long, 10 × 12 *μ*m^2^ rectangular cross section—1 mm long broadening to 10 × 50 *μ*m^2^ rectangular cross section—4 mm, 10 × 12 *μ*m^2^ rectangular cross section—1 mm long broadening to 10 × 50 *μ*m^2^ rectangular cross section—1 mm broadening to 10 × 100 *μ*m^2^ rectangular cross section—3 mm, 10 × 25 *μ*m^2^ rectangular cross section—1 mm long broadening to 10 × 50 *μ*m^2^ rectangular cross section—4 mm, 10 × 25 *μ*m^2^ rectangular cross section—1 mm long broadening to 10 × 100 *μ*m^2^ rectangular cross section—4 mm, 10 × 50 *μ*m^2^ rectangular cross section—1 mm long broadening to 10 × 100 *μ*m^2^ rectangular cross section—4 mm	*Q* = 5–20 *μ*l/h	70
Cells—red blood cells	N/A	75 *μ*m ID circular cross section	*Re* = 0.004–0.005	139
Cells—red blood cells	N/A	50 *μ*m ID circular cross section, 100 *μ*m ID circular cross section	*Re* = 0.003–0.005	135
Platelets	2.5	5000 × 100 *μ*m^2^ rectangular cross section—250 mm long	*Re* = 0.21–0.60	154

It has been found that larger cells migrate more quickly to equilibrium positions than their smaller counterparts^82,134^ and larger cells also migrate more in the direction of the channel center.^20,71^ Furthermore, it has been demonstrated, by both this group and others, that $F_{D}$ can cause cells with a lower E to migrate toward the center of the channel, while stiffer cells are more evenly distributed across the channel width.^20,21,23,81,145^ It has also recently been found that deformable cells travel in microchannels at higher velocities than rigid particles, which may be a result of deformable cells migrating toward the channel center.^146^ Many current microfluidic devices exploit these described inertial effects due to both cell size and deformability in order to separate mixed cell solutions (see Sec. V B).

#### 4. Computational studies

Investigations on the inertial migration of both solid and deformable particles are not only confined to experimental setups, but have also been studied computationally. Many studies have examined the migration of rigid particles in a wide variety of environments, including square and circular channels, Newtonian and non-Newtonian fluids, as well as different Re.^37,45,46,48,54,140,147–150^ Of particular interest are those investigating the effects of particle shape. It has previously been shown that the shape of rigid particles can influence their migration patterns in a microchannel.^144,151^ Notably, it has been shown that centerline-focusing particles typically have fore-aft asymmetry characteristics, similar to “fish” or “bottle’-like shapes.^151^

Further computational studies, which have examined the same reactions in deformable particles, have used this technique to model the behavior of RBCs^152,153^ and others. Magnaudet *et al.*^56^ found that the deformability-induced lift can be attributed to the surface tension gradients at the interface between the fluid and particle surface. Furthermore, it was found that this deformability also heavily influences its migration speed, with more deformable particles reaching equilibrium positions quicker.^31^ Additionally, despite the strong influence that Re has on the inertial positions of rigid particles, computational studies have also shown that Re has little to no impact on the migration of deformable particles, or cells, in circular channels.^31^

### C. Cell adhesion

Previous studies examining cell adhesion have demonstrated that the expression of signaling factors can enhance the process. In the LS for example, vascular endothelial growth factor C (VEGF-C), expressed by lymphatic endothelial cells and primarily responsible for lymphangiogenesis, has been associated with increased CTC metastasis,^155–160^ while activated chemokine receptor CCR7, expressed by T and B lymphocytes, causes cells to migrate toward lymphatic endothelial cells expressing the ligand CCL21.^161,162^ In addition, fluidic forces present in the channel can also affect cellular adhesion to the vessel walls, playing a part in both WBC and CTC adhesion.

In WBCs, previous investigations found that shear-induced cell deformation increases the surface area between a WBC and the endothelial cell layer, increasing its adhesive potential.^163^ Furthermore, WBCs, which have adhered to the endothelial layer, experience a sharp increase in $\nabla \tau_{p}$ when they do so.^164^ It was shown that while WBCs require a certain level of $\tau_{w}$ for adhesion to occur,^72,165^ beyond a threshold level (approximately 0.1 Pa), high $\tau_{w}$ can cause swelling in the cells and decreased cell stiffness, leading to a potential lack of integrin anchoring.^166,167^ In addition to the flow conditions, the channel geometry also significantly affects cell adhesion with bond formation much more likely in curved channels than straight.^168^

Despite the wealth of knowledge available on WBC adhesion and extravasation through endothelial cell layers, the same behavior in CTCs is not understood to a similar extent.^165^ In order for invasion to occur, the cell must stop or be stopped in its journey through the vessel. Small vessels can halt the progression of CTCs by physical occlusion if the vessel diameter is smaller than that of the cell’s diameter (approximately 10 μm).^86^ This has been observed by Kienast *et al.*^169^ to occur in the brains of mice where the blood vessels remain at quite small sizes. Having said this, larger vessel diameter has also been shown to increase CTC invasion.^99^ Furthermore, it has been found in larger vessels that

$$P_{A}\propto {\;f}_{c}t,$$ (26)

where $P_{A}$ is the probability of CTC arrest, $f_{c}$ is the frequency of collision between endothelial ligands and membrane-bound receptors, and t is the residence time.^103,168^ Collisions with RBCs have also been found to influence the trajectories of CTCs.^170^ Besides the size of the vessel, its shape also has a significant effect. Like WBCs, it has been found that circulating cells are more likely to adhere to the blood vessel wall of a curved vessel, rather than a straight one. This theory was first looked at in thrombi by Liu *et al.*;^171^ however, it was expanded later to include CTCs with further experimental and computational fluid dynamics (CFD) studies (using the Lattice–Boltzmann method) proving this theory.^168,172^ Furthermore, Yan *et al.*^172^ showed that the rate of adhesion is 1.5 times more likely in curved vessels than in straight ones. Bifurcations also increase the likelihood of tumor extravasation.^108^

Vessel fluid mechanics can also play a significant role. Circulation patterns can influence the direction that CTCs are carried, and consequently, their final destination.^86^ It has been observed that an increase in the fluid flow rate increases the cell adhesion in a blood vessel. It is believed that this is due to an increase in bond formation [or an increase in $f_{c}$ in Eq. (26)], as a high flow rate increases the chances of interaction between the CTCs and surrounding particles in the blood.^167^ Contrasting studies have found a link between decreased flow velocities and increased cell arrest.^173^ In later studies, similar to WBCs, it has been found that $\tau_{w}$ also has an effect on the adherence of CTCs to the vessel wall. Both CFD and experimental studies have shown that once a certain $\tau_{w}$ threshold has been reached in the vessel, bond association and disassociation rates may change, causing the cells to activate certain receptors and, consequently, become more likely to adhere to the blood vessel walls^172,174,175^ and to extravasate.^117^ It was concluded that unless the $\tau_{w}$ reaches this level, its effects can be disregarded; however, this specific $\tau_{w}$ level is relatively low in comparison to the whole system.^1^ In further studies by Mina *et al.*,^111^ that utilized microfluidic devices to investigate the effects of τ(r) on breast cancer cells in a 3D culture, it was found that lower levels of τ(r) (approximately 0.1 Pa) in the fluid, leads to increased breast cancer cell invasion in the CS. Again, though this is a relatively low $\tau_{w}$ to be found in the CS, it is quite high in comparison to the levels found in the LS, implying that $\tau_{w}$ levels in the lymphatics and veins^86^ are optimal for cancer metastasis. Further studies have shown, however, that increases in $\tau_{w}$ beyond this level (to approximately 3 Pa), may result in decreased cell adhesion to the endothelial cell layer.^108,176^ It must also be considered that while a small $\tau_{w}$ of approximately 0.1 Pa increases the adhesive capabilities of the CTC, it can also decrease its migration capabilities.^102^ Laminar τ(r) acting on a CTC can also cause the cell to enter G${}_{2}$/M arrest, thus inhibiting cell metastasis, while disturbed shear patterns are hypothesized to have the opposite effect.^177^ Additionally, the shear rate has been hypothesized to have a more significant effect on cell adhesion than $\tau_{w}$. Slattery *et al.*^178^ found that changes in the shear rate, even more than $\tau_{w}$, can cause a cell to become more partial to expressing binding molecules, causing it to adhere to the vessel wall.

Finally, differences in the metastatic capabilities between CTCs themselves can influence their adherence to a vessel wall.^179^ It is known that highly metastatic CTCs form stronger bonds to the endothelial cell layer than their more benign counterparts;^165^ they also have a much higher adhesion rate than less aggressive CTCs.^180^

## IV. THE ENVIRONMENT OF CELL SUSPENSIONS: *IN VIVO*

Suspended cells in the circulation can be exposed to a wide range of fluidic environments: from the interstitial environment where fluid surrounding cells is not constrained and flows at very low velocities, to the largest arteries which resemble very high-velocity, pulsatile pipe flows. The primary sites of microfluidic flow in the body occur in the lymphatic and cardiovascular capillaries. A comparison of the LS and CS is summarized in Table VI.

**TABLE VI.** Comparison of the cardiovascular and lymphatic systems.

Characteristic	Cardiovascular system	Lymphatic system
Capillary size	5–10 *μ*m	100–300 *μ*m
Fluid *μ*	Shear thinning fluid	Newtonian, 1 mPa s
Fluid *ρ*	1060 kg/m^3^	1000 kg/m^3^
Fluid Velocity	≤300 mm/s	0.35–1 mm/s
*Re*	1–4000	<1
*τ*_*w*_	1.5–60 Pa	0.065 Pa
$\nabla \tau_{p}$	0.004–0.023 Pa/*μ*m	0.004–0.137 Pa/*μ*m

### A. The cardiovascular system

The cycle of the CS, the more well known and extensively researched system, begins in the heart, when oxygenated blood is pumped from the heart, via the aorta, to the rest of the body. From here, arteries carry oxygenated blood to smaller arterioles and finally, blood capillaries. The capillaries lie among the cells of the tissue and secrete and absorb the interstitial fluid surrounding the cells. It is here that nutrient and waste exchange takes place and excess interstitial fluid is later absorbed by the lymphatic capillaries (see Sec. IV B).^181^ Blood capillaries are also extremely small at around 5–10 μm. Fluid flows through these capillaries by means of a pressure difference (high pressure in arteries to low pressure in veins) and fluid exchange in and out of the capillaries is governed by the revised Starling principle,^182^

$$\frac{J_{v}}{A}=L_{m}((P_{c}-P_{i})-\sigma_{s}(\Pi_{p}-\Pi_{i})),$$ (27)

where $J_{v}$ is the volume filtration rate per unit endothelial area A, $L_{m}$ is the hydraulic conductivity of the membrane, $P_{c}$ is the capillary hydrostatic pressure, $P_{i}$, the interstitial hydrostatic pressure, $\sigma_{s}$ is Staverman’s reflection coefficient, $\Pi_{p}$ is the osmotic pressure in plasma, and $\Pi_{i}$, the osmotic pressure in the interstitial fluid.^183^ Blood leaving the blood capillaries travels through venules, veins, and, finally, back to the heart.

Because of the high pressure exerted by the heart, fluid velocities in the CS can reach up to 300 mm/s. Blood is a non-Newtonian, shear thinning fluid with a density slightly higher than water of approximately 1060 kg/m${}^{3}$; however, at the scale of capillaries, it is treated discretely. Due to the different flow regimes in arteries, veins, or capillaries, the Re in the CS varies greatly (approximately 1–4000), and so fluctuates between laminar and turbulent flow. These result in a $\tau_{w}$ in the CS of 1.5–60 Pa,^6^ resulting in $\nabla \tau_{p}$ of approximately 0.004–0.023 Pa/μm.

### B. The lymphatic system

The LS, like the CS, is a circulatory system; however, unlike the CS, the LS is an open system, and lymph does not remain exclusively within the vessels.

The lymph’s journey begins in the lymphatic capillaries, or the initial lymphatics. These channels are found lying among the cells and blood capillaries and contain tiny valves which open by means of a pressure difference, absorbing the interstitial fluid. From here, the interstitial fluid becomes known as lymph.^184^ The lymphatic capillaries, like blood capillaries, are only one cell thick; however, unlike blood capillaries, their shape tends to be inconsistent with a diameter ranging from 10 to 60 μm.^185,186^ Following its passage through the capillaries, lymph flows into the collecting lymphatics, whose walls are much thicker, more muscular, and have a larger diameter (approximately 100–300 μm).^184,187–191^ Instead of a pressure difference, at this stage, the LS employs a number of different techniques in order to transport the lymph. Muscular walls of the lymphatics can contract, squeezing the lymph slowly along.^184,192^ These muscles act independently of each other, their responses depend upon the local fluid dynamics they are normally exposed to.^193^ Therefore, different vessels, depending on their positions in the body, respond differently to changing levels of intra-luminal pressure.^194^ Muscular contraction of larger muscles, such as biceps and triceps, also squeezes the lymphatics, causing the lymph to be pushed forward.^184,192^ Finally, the lymphatics contain valves, similar to veins. These prevent the back-flow of lymph and ensure it remains moving forward.^195–197^ The valves are biased to stay open in order to allow efficient pumping;^197,198^ however, should the situation require, they will close. The vessel between two valves is known as a lymphangion.^199^ From the collecting lymphatics, the lymph is taken via the lymphatic ducts and returned to the blood at the subclavian veins in the shoulders.

Because of the passive nature of the LS, fluid velocities are much lower in comparison to the CS (0.35–1 mm/s).^188–191^ Additionally, lymph is considered to be a Newtonian fluid with a dynamic viscosity and density similar to those of water (approximately 1 mPa s and 1000 kg/m${}^{3}$ respectively).^189,200,201^ Each of these individual factors combines to give lymphatic fluid flow a very low Re, typically, Re<1.^187^ Larger capillaries and lower velocities also result in a lower $\tau_{w}$ in the LS of approximately 0.065 Pa,^187,189,190^ and previous studies have shown that Poiseuille flow is a valid assumption for estimation of $\tau_{w}$ in lymphatic flow.^189^ Interestingly, previous computational studies have also found that $\nabla \tau_{p}$ in the LS are quite large in comparison to the CS, reaching values of 0.004–0.137 Pa/μm.^202^

## V. THE ENVIRONMENT OF CELL SUSPENSIONS: *IN VITRO*

*In vitro* environments, while not the native conditions of circulating cells, are more straightforward in terms of fluid mechanics. These applications tend to revert to Newtonian fluids and undergo laminar flow in straight, regular microchannels. Cells are exposed to *in vitro* flow conditions for research, diagnostic and treatment purposes. These applications are expanded further below.

### A. Lab-on-a-chip devices

Lab-on-a-chip devices or organ-on-chip-devices primarily replicate *in vivo* tissue conditions allowing for the study of specific organs in healthy or diseased states in a controlled, biologically accurate environment, which can also be used for researching therapeutic applications.^203^ Many imitate cancer tissue and can be used to replicate and study cancer metastasis,^204–206^ examining cells’ migration through the extracellular matrix (ECM). Others can be fabricated in order to resemble vascular microvessels.^207–210^ These vessels, however, are primarily used to either examine the response of adherent endothelial cells to fluidic forces^211–213^ and nanoparticle interactions,^214^ or the process of cancer migration and intravasation^215–219^ in blood vessels. Some examine the same phenomena in lymphatic vessels.^220,221^ However, the activities of suspended cells already flowing within the channel are, by comparison, less well understood. One of the few to study this, Follain *et al.*,^173^ found that lower flow velocities were favored by CTCs for cell arrest. As different devices are developed to investigate different biological phenomena, the magnitude and type of fluidic forces that cells experience are unique to each assay. For this reason, cell reactions to fluidic conditions may be particularly difficult to establish for lab-on-a-chip devices without preliminary tests. Cell viability is important in these devices as, in the case of therapeutic applications, it is imperative that cell damage or death can be attributed to the variable under consideration and is unrelated to the fluidic forces that the cells may experience in the microfluidic device.

### B. Cell separation

The majority of applications in the inertial migration of deformable particles lie in particle or cell sorting. Numerous devices have been developed in order to separate different particles from each other based on size, deformability, or surface markers. The majority of these applications lie in the biomedical industry, with the separation of different cell types from blood in particular. Cell separation is often necessary for diagnostic purposes when one cell type alone is required or, like the case of CTCs, cell enrichment is requisite for rare cells.

#### 1. Inertial cell separation

Inertial cell separation techniques utilize micrometer-sized channels in order to manipulate the inertial forces acting on cells, allowing them to be separated based on different physical parameters.^222^ Studies that have developed this technique have been summarized in Table VII along with particle separation techniques. Cell separation differs from other *in vitro* cell suspension applications in that, often, cells are exposed to larger viscous drag forces or Dean forces (see Sec. II B 2 5) within the channel,^97^ increasing the risk of cell damage. Previous studies have found that WBC solutions in a spiral microchannel, similar to those used in cell sorting applications, can induce cell losses of up to 10.3% and cell deformation of up to 29.8%.^24^ Again, cell viability and functionality is essential for cell separation applications, as the properties of cells will change upon cellular damage or death, resulting in potential faulty separation.

**TABLE VII.** Summary of the developed particle inertial separation techniques.

Separated particle(s) (*d*_*p*_)	Separated from	Channel description	*Re*/*Q*	Study
Spherical particles (15.5 *μ*m), spherical particles (18.7 *μ*m), spherical particles (26.3 *μ*m), spherical particles (31.2 *μ*m), white blood cells (≥7 *μ*m)	PBS, whole blood	45 × 120 *μ*m^2^ rectangular cross section sheath flow (particles), 50 × 150 *μ*m^2^ rectangular cross section sheath flow—20 mm long (cells)	*Q*_*Blood*_ = *Q*_*PBS*_ = 112.5 *μ*l/min (particles), *Q*_*Blood*_ = 133 *μ*l/min *Q*_*PBS*_ = 267 *μ*l/min (cells)	225
PDMS particles (20 *μ*m), lung cancer cells (NSCLC) (11–27 *μ*m), prostate cancer cells (VCaP) (12–35 *μ*m), breast cancer cells (MDA-MB-231) (12–29 *μ*m)	PBS, whole blood	Vortex chip: 70 × 40 *μ*m^2^ rectangular cross section broadening to ×8 reservoirs in series with ×8 channels in parallel	*Re* = 150–160	83
Particles (6 *μ*m), particles (15 *μ*m), breast cancer cells (MCF-7) (15–20 *μ*m), breast cancer cells (MDA-MB-231) (15–20 *μ*m), cervical cancer cells (HeLa) (15–20 *μ*m)	PBS, whole blood	160 × 500 *μ*m^2^ rectangular cross section—10 cm long in a two-loop spiral	*Re* ≈ 50	63
Polystyrene particles (1 *μ*m), polystyrene particles (4.8 *μ*m)	Water	75 *μ*m ID circular cross section—5–60 cm long	*Re* = 5.6–28	226
Polystyrene particles (7.32 *μ*m), polystyrene particles (10 *μ*m), polystyrene particles (15 *μ*m), polystyrene particles (20 *μ*m), blood cells	Water, whole blood	75 × 250 *μ*m^2^ rectangular cross section—6 cm long in a four-loop spiral, 110 × 500 *μ*m^2^ rectangular cross section—8 cm long in a four-loop spiral	*Q* = 1–3 ml/min	64
Polystyrene particles (5 *μ*m), polystyrene particles (15 *μ*m), cervical cancer cells (HeLa) (13 *μ*m)	Water, whole blood	85 × 300 *μ*m^2^ rectangular cross section—334 mm long in a six-loop double spiral	*Re* = 18–106	65
Particles (9.94 *μ*m), particles (20 *μ*m), prostate cancer cells (18–22 *μ*m)	Water, whole blood	50 × 27 *μ*m^2^ rectangular cross section—10.3 mm long broadening to 50 × 100 *μ*m^2^ rectangular cross section—9.2 mm long	*Re* = 30–80	66
Polystyrene particles (3 *μ*m), polystyrene particles (6 *μ*m), polystyrene rods (AR:1:3), polystyrene rods (AR:1:5)	Water	47 × 25 *μ*m^2^ rectangular cross section—4 cm long, 47 × 30 *μ*m^2^ rectangular cross section—4 cm long, 47 × 35 *μ*m^2^ rectangular cross section—4 cm long	*Re* = 13–72	227
Breast cancer cells (MCF-7) (18.1 *μ*m), breast cancer cells (MDA-MB-231) (18.2 *μ*m)	PBS	20 *μ*m wide rectangular cross section—100 *μ*m long broadening to 60 *μ*m wide rectangular cross section—100 *μ*m long with ×75 reservoirs in series	*Re* = 10–150	80
PDMS particles (2–30 *μ*m), oil droplets (6-20 *μ*m), leukocytes, cervical cancer cells (HeLa), breast cancer cells (MCF-7), bone cancer cells (SAOS-2)	Water, whole blood	93 × 40 *μ*m^2^ rectangular cross section—4.5 cm long broadening to five branched outlets	*Re* = 21–42	23
Polystyrene particles (1 *μ*m), polystyrene particles (4.8 *μ*m), polystyrene particles (9.9 *μ*m), cervical cancer cells (HeLa) (12.4 *μ*m), Breast cancer cells (MCF-7) (20 *μ*m)	Water, whole blood	70 × 50 *μ*m^2^ rectangular cross section with ×10 reservoirs broadening to 70 × 400 *μ*m^2^ rectangular cross section—400 *μ*m^2^ long in series with ×8 channels in parallel	*Re* = 5–270	81
Polystyrene particles (6 *μ*m), neuroblastoma cells (SH-SY5Y) (15 *μ*m)	Water, PBS	50 × 100 *μ*m^2^ rectangular cross section—25 cm long in a ten-loop spiral, 120 × 500 *μ*m^2^ rectangular cross section—40 cm long in a five-loop spiral	*Re* ≈ 20	224
Polystyrene particles (3 *μ*m), polystyrene particles (6 *μ*m), red blood cells (approximately 6 *μ*m), malaria-infected red blood cells (approximately 3 *μ*m)	Whole blood	10 × 15 *μ*m^2^ rectangular cross section—3 cm long with three outlets	*Q* = 0.2–5 *μ*l/min	13
Polystyrene particles (7.9 *μ*m), red blood cells (5–12 *μ*m), bacteria (*E. Coli* K-12) (1–3 *μ*m)	Water, whole blood	60 × 20 *μ*m^2^ rectangular cross section—4 mm long broadening at 0.2^°^ to 60 × 160 *μ*m^2^ rectangular cross section with three outlets	*Re* = 17–84	62
Polystyrene particles (2.4 *μ*m), polystyrene particles (5.9 *μ*m)	Glycerin solution	50 *μ*m^2^ square cross section	*Re* = 0–0.37	228
Polystyrene particles (10 *μ*m), polystyrene particles (15 *μ*m), polystyrene particles (20 *μ*m), neuroblastoma cells (SH-SY5Y) (15 *μ*m), rat glioma cells (C6) (8 *μ*m)	Water, PBS	90–140 × 500 *μ*m^2^ rectangular cross section in a six-loop spiral		229
Polystyrene particles (1.9 *μ*m), polystyrene particles (7.32 *μ*m)	Water	50 × 100 *μ*m^2^ rectangular cross section—13 cm long in a five-loop spiral	*Re* ≤ 10	47
Polystyrene particles (3.1 *μ*m), polystyrene particles (9 *μ*m), oil droplets (≤20 *μ*m), PDMS particles (≤20 *μ*m)	Water	50 × 350–650 *μ*m^2^ rectangular cross section, ellipse-shaped units 31 units in length	*Q* = 0.9 ml/min	230
Polystyrene particles (2 *μ*m), polystyrene particles (3 *μ*m), polystyrene particles (4 *μ*m), polystyrene particles (7 *μ*m), polystyrene particles (9 *μ*m), polystyrene particles (17 *μ*m), oil droplets (≤20 *μ*m), lung cancer cells (H1650)	PBS, whole blood	Ellipse-shaped units	*Re* = 0.075–225	38
Polystyrene particles (8.7 *μ*m), red blood cells (8-9 *μ*m)	Dextran, whole blood	75 *μ*m height rectangular cross section narrowing to 75 × 15–50 *μ*m^2^ rectangular cross section—50–300 *μ*m long broadening to 75 *μ*m height rectangular cross section	*Re* = 0.01	22
Polymer particles (0.71 *μ*m), polymer particles (1 *μ*m), polymer particles (2.1 *μ*m), polymer particles (3 *μ*m), polymer particles (5 *μ*m), blood cells (7-8 *μ*m)	Water, whole blood	Multichannel	*Q* = 20–1000 *μ*l/h	231

#### 2. Magnetic cell separation

Magnetic cell separation techniques exploit the surface proteins of a specific cell type, utilizing antibodies, affixed to the surface of magnetic beads, which then attach to the desired cell, causing them to attach to magnetic surfaces, such as the beads of a separation column or the walls of a surrounding magnet. This technique is commonly used for the separation of blood for research purposes. While the use of microchannels is not as common in this application, suspended cells are still exposed to complex forces, increasing the risk of cell damage. Cell viability and functionality are essential, as the properties of cells will change upon cellular damage or death, resulting in potentially inaccurate separation.

### C. Flow cytometry

Flow cytometry is a microfluidic method used to measure or identify cell characteristics by illumination of fluorescently tagged cells as they flow past a light source.^223,224^ It can be used for diagnosis of certain conditions by the physical properties (size or deformability), chemical properties (expression of proteins), or simply by the presence or absence of the cells. For example, fluorescence-activated cell sorting (FACS), counts or sorts cells by means of the presence or absence of a fluorescent antibody, which attaches to the surface of the cell due to the presence of a specific surface protein.^153^ Again, like the majority of flow cytometry techniques, a microchannel is used to expose the cell surface to a laser, producing a signal for cell analysis. This technique can be used as a standalone procedure or can be used in conjunction with cell separation techniques for faster processing. Within these channels, $\tau_{w}$ may reach values of up to 0.9–1.4 Pa. Like cell separation, it is important that both cell viability and functionality remain unaffected by the fluidic forces from the channel that the cells are traveling in, to prevent false diagnosis.

### D. Patient therapies

Microchannels are commonly used in clinical settings to extract cell suspensions from patients and return them to the body. Dialysis, used in patients with kidney failure to remove blood from the body, extract toxins and return it to the body, has been shown to disturb the local haemodynamics, leading to hyperplasia, stenosis, and ultimately, thrombosis of the endothelial cell lining.^232^ It is reasonable to assume that a similar damage may occur to suspended cells due to disrupted flow patterns. Similarly, apheresis, used in both donation (for example, blood plasma and platelets) and therapeutic purposes (for example, in leukemia or haemochromatosis) for isolation and removal of specific cell types from the blood, can induce disturbed local haemodynamics. Not only is there a change in the local fluid dynamics at the access points, but in both cases, cells are passed through microchannels at high velocities and, in the case of apheresis, may also undergo centrifugation, resulting in a variety of forces acting on the cell with a potential for cell damage. In these applications, the retention of cell viability and competence is paramount, as cells are returned to the body of the patient.

### E. Adoptive cell transfer

One of the most exciting and novel emerging areas of cancer research, adoptive cell transfer (ACT), promises to change the way cancer and autoimmune diseases are conventionally treated. Autologous cancer immunotherapy involves the removal of T-cells from the patient’s body, followed by modification and a return to the body of the same patient, reducing the risk of a foreign body response, while allogenic therapies involve separate donor and patient, advantageous in cases where patient cell counts have already been severely blighted.^233^ In early studies, tumor-infiltrating lymphocytes (TILs) were extracted from patients’ excised tumors, cultured *in vitro*, and returned to the patient’s body to treat melanoma.^234^ Antigen-expanded T-cell therapy operates on a similar principle; however, extracted T-cells are reactivated to recognize specific tumor-associated antigens, while T-cell receptor (TCR) therapy and CAR T-cell therapy involve the genetic reprograming of killer T-cells from the cancer patient to express T-cell receptors and chimeric-antigen receptor, respectively, before being returned to the patient’s body.^234,235^ ACT processing steps *in vitro* involve blood extraction, followed by cell separation techniques, and flow cytometry as previously described, and so, make use of microchannels. As the cells are being harvested from a patient with precious few T-cells that may already be quite weak due to patient treatment, it is imperative that the cells are not impaired further in any way by the T-cell extraction and treatment process. Additionally, as the patient has an already severely compromised immune system, it is critical to ensure certainty that returned T-cells are not impaired or damaged in any way by the forces that they are exposed to in these channels.

## VI. CONCLUSIONS AND FUTURE PERSPECTIVES

Currently, there is an extensive understanding of the advection of rigid particles in microchannels. A level of complexity is added, however, when deformable particles are used, particularly when the deformable particles are living cells, whose functionality and viability can also be impacted by the same fluidic forces that dictate their location in the channel.

While previous studies have attempted to assess the effect that these forces have on the viability and functionality of the cell, it is still difficult to ascertain the validity of these results as it is difficult to quantify these forces or to understand, which individual forces contribute to such effects. This is because test methods which are currently used to apply shear stresses to cell suspensions cannot replicate Poiseuille flow (cone and plate), can be time dependent (syringe and needle) or exert additional unknown strains on the suspended cells (continuous flow circuits). Additionally, in the latter two methods, it is difficult to ascertain the shear stress that cells in suspension are actually exposed to. For this reason, there is a need to develop a test method that will not only apply a continuous, non-compressive shear force to suspended cells in pipe flow over an extended period of time but also incorporate a method that will allow for a more accurate estimation of the shear forces imposed on the cells than the $\tau_{w}$ value.

From the conducted review, it is clear that there are significant gaps in the current knowledge on cells in suspension that require further study. While a number of studies have examined the inertial migration of particles and cells in rigid microchannels, to the authors knowledge, none have looked at the same effects in deformable channels. Future studies in deformable channels will aid us in the understanding of cell advection in the capillaries, potentially furthering our knowledge of nutrient exchange or cancer metastasis, and could be extended further to incorporate similar studies in excised vessels. In contrast, the majority of viability studies in circulatory models examine this outcome only in deformable microchannels and have not compared these results to the viability in rigid-walled channels. This makes it difficult to estimate the viability of these cells in certain *in vitro* channels, as the native environment of these cells being one with deformable channel walls may result in reduced viability outside of these situations.

Additionally, while it is evident from the published literature that a change in deformability results in a change in inertial migration, with a possible change in cell viability rates, the extent of the influence of the deformability change is unclear. Indeed, currently many cell separation devices already rely on this phenomenon that different cells occupy different inertial positions due to their size or deformability. However, it is still unknown if these are the only physical properties that influence a cell’s position. Further experimentation and computational work will confirm this.

This leads to uncertainty in microfluidic therapeutics, diagnostics, and research as unquantified forces, such as the shear stress on the cell surface, or the shear rate that the cell is exposed to, may be affecting the cell in an unknown capacity, potentially giving false negatives in the case of diagnostics or, in the case of therapeutics, resulting in the further depletion of an already compromised and weak cell source.

For this reason, it is vital that these effects are not only identified but also quantified in order to ensure the best outcomes for patients.

## Data Availability

Data sharing is not applicable to this article as no new data were created or analyzed in this study.
